# The secret life of RNA and lipids

**DOI:** 10.1080/15476286.2025.2526903

**Published:** 2025-07-04

**Authors:** Tomasz Czerniak, James P Saenz

**Affiliations:** B CUBE Center for Molecular Bioengineering, Technische Universität Dresden, Dresden, Germany

**Keywords:** RNA-lipid, RNA, lipid, RNA activity, RNA World

## Abstract

There is no life without RNA or lipids. But could there be life with only RNA and lipids? The discovery that RNA can catalyse reactions in addition to encoding information opened new directions for engineering life and the possibility of life emerging from an RNA World. But a key missing ingredient for RNA-based biochemical systems is a mechanism to organize RNAs and regulate their activity. Lipids, which are essential for life and one of the most ancient biomolecules, can spontaneously self-assemble to form membranous bilayers, theoretically providing a surface that can serve to concentrate, protect, and regulate RNAs. This review explores the interactions between RNA and lipids, including the chemical basis for their interactions, and the implications for synthetic biology, RNA World, and modern cell biology. We discuss observations that RNA can selectively bind to lipid membranes in a sequence-dependent manner, and entertain how these interactions might be employed to engineer RNA-based sensors and regulatory elements in synthetic systems. The emerging field of RNA–lipid interactions opens new possibilities for engineering orthogonal biochemistries for synthetic cells, innovations in RNA therapeutics, and discovering potentially new facets of cellular regulation.

## GRAPHICAL ABSTRACT

**Figure:**
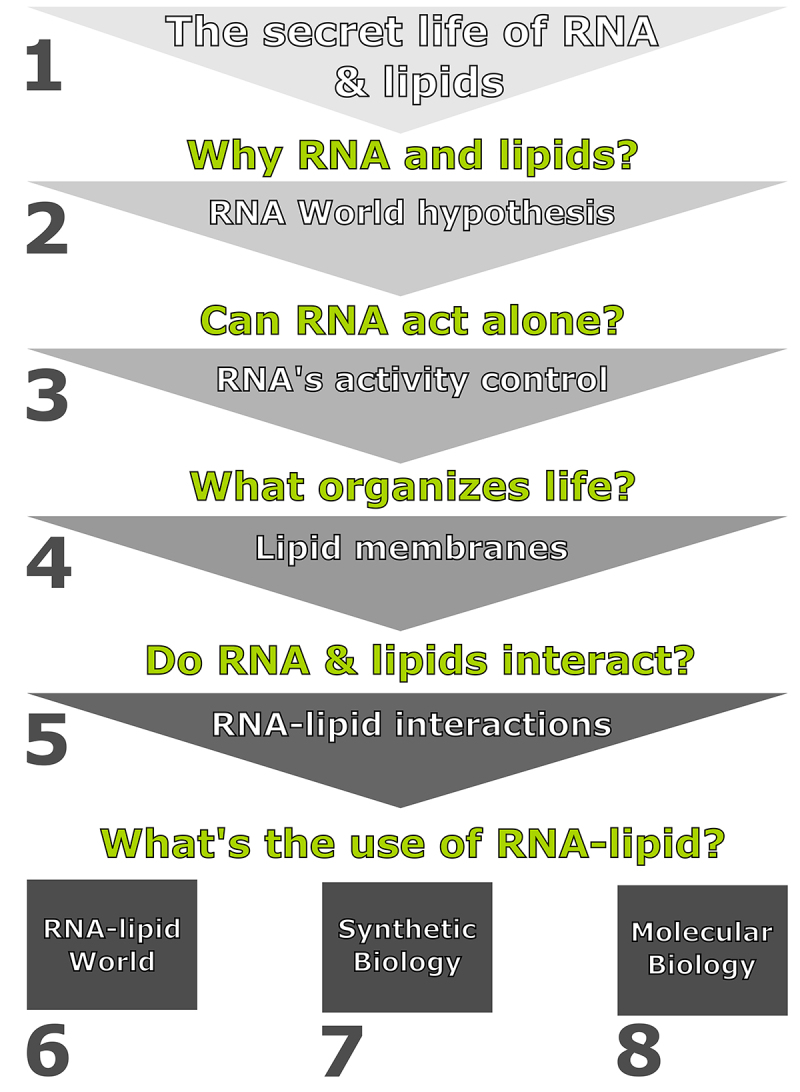

## 1. Introduction

RNA can serve diverse roles including catalyst (e.g. ribozymes), regulator (e.g. riboswitches), and information carrier (e.g. mRNA), giving it central importance in the origin and function of cellular life. The first discovery of catalytic RNAs inspired the RNA World hypothesis that living systems could have emerged from entirely RNA-based biochemical systems [1]. Although we may never know if life started with an RNA World, the concept has spawned decades of research demonstrating that catalytic and regulatory RNAs can be synthetically evolved from large pools of random RNA sequences. The diversity of functions that can be performed by RNA alone is increasing and may eventually approach the complexity required for assembling synthetic RNA-based life. Indeed, for synthetic biology the prospect of RNA-based synthetic systems that do not require complex transcription and translation machineries has great utility. Moreover, the synthetic RNA World provides an experimental platform for studying the principles underlying the evolution of function in a biochemically naïve context [2,3]. However, a key requirement for life-like synthetic RNA systems is organization in space and time. But how can RNA be localized and regulated in a simple synthetic system, or in a prebiotic environment? One of the prebiotically plausible molecules which is present as well in all living cells are lipids which can spontaneously self-assemble to form membranous bilayers, theoretically providing a scaffold that can serve to concentrate, protect, and regulate RNAs [4].

It was recently demonstrated that RNA can interact directly with lipids in a sequence-dependent manner, and that this interaction changes ribozyme activity [5,6]. This work builds on a handful of studies over the past several decades revealing that RNA can interact directly with zwitterionic, positively or negatively charged lipid membranes [7–9]. Ours and others observations indicate that RNA–lipid interactions can be selective for membrane property or lipid composition [10–12], providing a basis to engineer RNAs as membrane property sensors, or to selectively localize functional RNAs in synthetic or cellular systems. In the light of the recent global importance of lipids in the delivery of mRNA vaccines, insights into the selectivity and stability of interactions between RNA and physiologically relevant lipids are of great value to bioengineering and medicine. Further, there is some evidence suggesting that RNA can selectively alter the properties of lipid membranes [10–14], giving an impetus to study how RNA can be used to remodel membrane properties, such as curvature, permeability and lipid order. These observations raise the possibility that selective RNA–lipid interactions could be involved in cellular regulation, and provide a simple answer in the debate on the RNA World scenario for the origin of life: how could RNA systems be organized in a prebiotic environment [15]?

**Figure 1.**
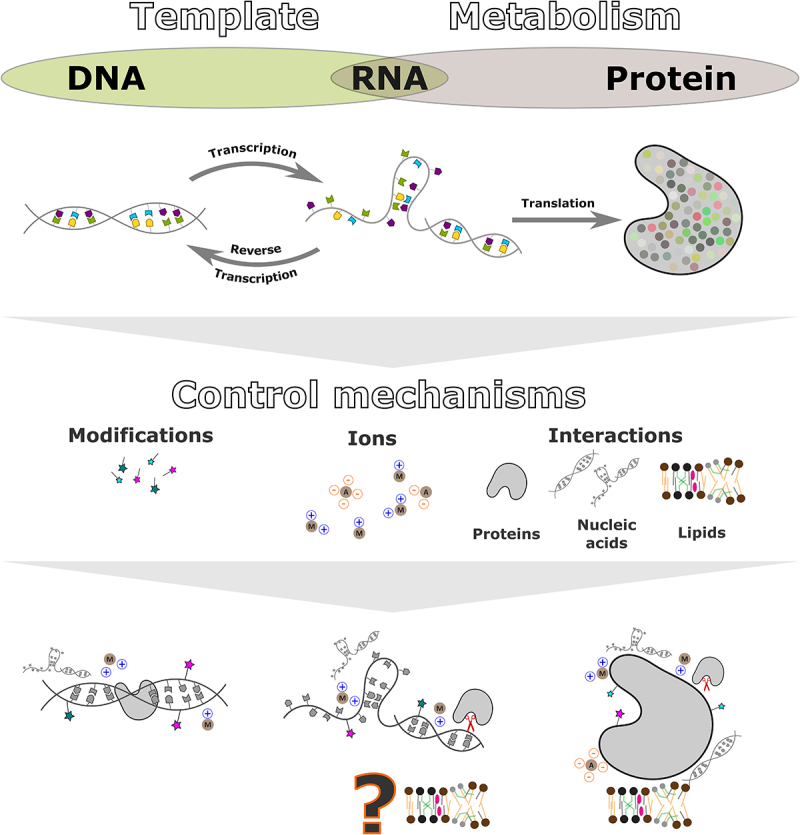
Premise for an RNA–lipid world. The central dogma of molecular biology defines DNA as the template to produce RNA (transcription), which then serves as a template to produce proteins (translation) or DNA (reverse transcription). Proteins have various activities that support life (metabolism). RNA can also participate in metabolic activities (ribozymes, riboswitches, aptamers), making it a unique molecule capable of both storing heritable information and catalysing biochemical reactions. Most biomolecules require various regulatory mechanisms to maintain their essential functions for life (e.g. chemical modifications, interactions with ions, and other biomolecules). Nucleic acids, for example, can be regulated through epigenetic or post-transcriptional modifications (for DNA and RNA, respectively), interactions with proteins (e.g. topoisomerases, nucleases), and interactions with other nucleic acids. Protein activity can be controlled via a large matrix of interactions and modifications, in which interactions with lipid membranes play a vital role (more than 25% of the human genome encodes lipid membrane proteins [16]. Interestingly, current research suggests that lipid-dependent regulation of RNA activity may also be an important factor in maintaining life.

In this review, we discuss the current insights on RNA–lipid interactions (Figure 1). We describe the known RNA–lipid binding mechanisms (determined experimentally and *in silico*) and their effect on both binding partners. Further, we speculate on the plausible scenarios in which RNA–lipid interactions could be beneficial. In particular, we will discuss RNA–lipid interactions in the context of prebiotic biochemistry (RNA–lipid World), synthetic biology (artificial cells based on the interplay of RNA and lipids), and molecular biology (RNA–lipid interactions *in vivo*).

## 2. The RNA World hypothesis

The diverse range of RNA functions makes them an attractive subject in prebiotic biochemistry research. Information storage, ribozyme, riboswitch, and aptamer-based activities can, in principle, form a robust and responsive basis for the biochemistry of early protocells. This has led several authors to suggest that a so-called ‘RNA World’ could have emerged. The first suggestion of RNA being a vital part of the prebiotic world was coined by Alexander Rich in 1962 – shortly after linking DNA and RNA molecules with the expression of genetic information, and many years before description of the first ribozymes, aptamers, and riboswitches [17]. This elegant thought experiment explains the plausible scenarios which might have led to creation of first self-replicating systems with nucleic-acid-based templating mechanisms without the involvement of proteins. 20 years later, after discovery of the first ribozymes [18–20] it became plausible to develop the RNA-first-based hypotheses into the much richer RNA World hypothesis [1].

**Figure 2.**
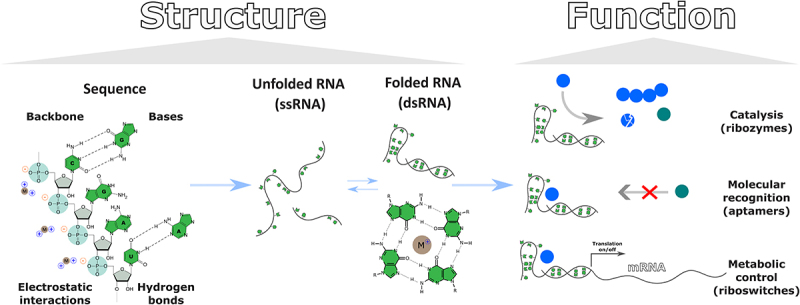
RNA structure underlies its functionality. RNA, through the interaction with ions and base pairing, forms various structures. The combination of single stranded (unfolded RNA, loops) and double stranded (A-type double helix, G-quadruplexes, pseudoknots) RNA structures can form complex assemblies in which RNA can interact with different binding partners. The recognition of different molecular targets leads to complex activity patterns such as catalysis (ribozymes), translation control (riboswitches), and molecular organization (aptamers).

RNA is a biopolymer composed of nucleotides linked by phosphodiester bonds (Figure 2). Typically, each nucleotide contains phosphate, sugar (ribose), and nucleic acid base (guanine, G; adenine, A; cytosine, C; uracil, U). RNA’s negatively charged phosphate backbone has the affinity to bind positively charged cations, such as Mg^2+^, Ca^2+^, K^+^ etc [21]. On the other hand, nucleic acid bases can form hydrogen bonds with other nucleic acid bases forming the double stranded RNA which further can create more complex structures. Alternatively, nucleic acid bases can also bind ions [22,23]. The RNA structure, i.e. position of ions, base pairing, including both canonical A-U and G-C and non-canonical structures (for example, Hoogsteen interactions, GU wobbles, G-quadruplexes [24,25]) directly translates into the functionality and the potential of interacting with different binding partners (if the RNA is correctly folded [26–28]).

Is it possible that RNA molecules were present in the prebiotic world? A series of experiments proved that, under the right conditions, nucleic acid monomers can be spontaneously synthesized [29,30]. Moreover, spontaneous polymerization of RNA monomers can occur, creating oligomers up to 30 nucleotides long [31]. Notably, lipids can enhance the polymerization process [32–34]. Additionally, it was described that under certain conditions RNA can ligate in aqueous solvent, which might lead to the presence of longer RNA species [35–40]. It was reported that RNA species shorter than 40 nt can have catalytic [41] or aptamer [42] activity, which creates the possibility of metabolically active RNA species in the RNA World as well.

Although the idea of assembling the first life-like systems solely based on RNA is appealing due to its simplicity, it is more plausible that, in the ‘prebiotic soup’, various molecules – such as ions, amino acids, lipids, and small solutes – played a role [43]. In this scenario, RNA would have interacted with these molecules, utilizing their properties to maintain its integrity and activity. For example, one could hypothesize that RNA initiated a primitive reverse citric acid cycle (rTCA) [44], producing various molecules through RNA aptamer and catalytic activity (overcoming the problem of rather unlikely spontaneous rTCA [45,46]), and those molecules contributed to further development of the protocellular life. In addition to the RNA World hypothesis, other theories have been proposed, including lipid-first, metabolism-first, protein-first, and thioester-first scenarios [47]. It is also possible that, before the emergence of life as we know it, different pre-life-like systems may have been created but ultimately failed to sustain themselves.

Numerous groups have explored the concept of RNA-based prebiotic systems [48–53]. Key findings contributing to the RNA World hypothesis involved the development of ribozymes and aptamers with a broad variety of protein-like activities. Theoretically, it is possible to maintain simple life-like systems using only RNA. The main limitations of these systems in the prebiotic world, however, are the lack of control over the RNA activity, the RNA-destructive conditions (for example the presence of the UV-light), and the lack of physical boundaries which could act as scaffolds and regulatory platforms for RNA species.

## 3. Can RNA act alone?

The classic point of view of RNA species as information carrier [54] has changed over the years, and now we have access to a broad variety of both naturally occurring and *in vitro* selected ribozymes and riboswitches with different activities, structures, and mechanisms, as well as RNA aptamers which can recognize a broad range of molecular targets. Such a great range of plausible RNA activities, structures, and functionalities is astonishingly diverse for a single type of molecule. As with all biomolecules, however, RNA activity has to be strictly controlled in order to preserve its correct function within a given system. In most cases, in order to be active, RNA biomolecules are regulated and organized through interactions with other molecules, such as proteins, metal ions, and other nucleic acids. For example, the stability of some small RNAs is strictly related to binding the Argonaute proteins (Argonaute proteins are responsible, for example, for the RNA-based control of the gene expression) [55]. Other examples of protein-based control of RNA activity include RNase P and ribosomes [56–58]. Activity of most of the RNA species is modified and controlled through the interaction with metal ions, especially magnesium and potassium cations; most ribozymes need metal ions to form stable and catalytically active RNA folds [59–62]. For example, some catalytic RNAs, such as HDV ribozyme, use metal ions in the reaction core as the charge transfer moiety [63]. On the other hand, the activity of hammerhead ribozymes depends on the presence of divalent ions, however it can be also supported in high monovalent ion concentrations [64,65]. There are indications of RNA interacting with anions as well [66]. Lastly, RNA interactions with other nucleic acids play a role in gene expression patterns and RNA stability [67,68]. Another remarkable example of RNA activity controlled by non-RNA binding partners are riboswitches and aptazymes; for instance, the activity of ligase ribozyme can be tuned by the presence of ATP or theophylline [69–71]. All of the aforementioned control mechanisms involve spatial organization, reaction orchestration, and structure modification of RNA species in a way similar to protein-based reactions. In the other words, the key for maintaining optimal activity of biomolecules is organization. One of the most prominent and evolutionarily conserved organizational platforms present in cells are lipid membranes [72,73]. There is plenty of evidence that biomolecules, especially proteins, can be controlled by lipid membranes in various ways (for example, the structure of the proteins can be controlled by different membrane composition and fluidity [74,75]). In principle, lipid membranes can interact with RNAs and act as an organizational scaffold. This adds weight to the argument that RNA can exhibit protein-like properties, which could have very important implications for the RNA prebiotic World hypotheses as well as in modern molecular biology.

## 4. Lipid membranes support life

Lipids, similar to RNA, are present in all living organisms and play a vital role in cell metabolism. There are a very broad variety of lipid species (e.g. unique chemical structures), which makes it challenging to systematically organize lipid types and classes. However, there are some general lipid features which are common for all types and classes.

Lipids have a hydrophobic or amphiphilic nature; therefore, they tend to spontaneously form aggregates in an aqueous environment and, depending on the structure of the lipid, those aggregates have different phenotypes. Hydrophobic lipids completely phase separate from aqueous solvent, which either creates solid aggregates or an oil-in-water emulsion. Amphiphilic lipids tend to create a variety of structures, in which the hydrophobic parts are separated from the water solvent, while the hydrophilic parts remain in solution. This kind of self-assembly is broadly known as the hydrophobic effect, and is primarily entropy-driven as it minimizes contact between water molecules and hydrophobic surfaces, thereby minimizing the system’s free energy. Thus, lipids tend to spontaneously self-assemble into stable aggregates.

**Figure 3.**
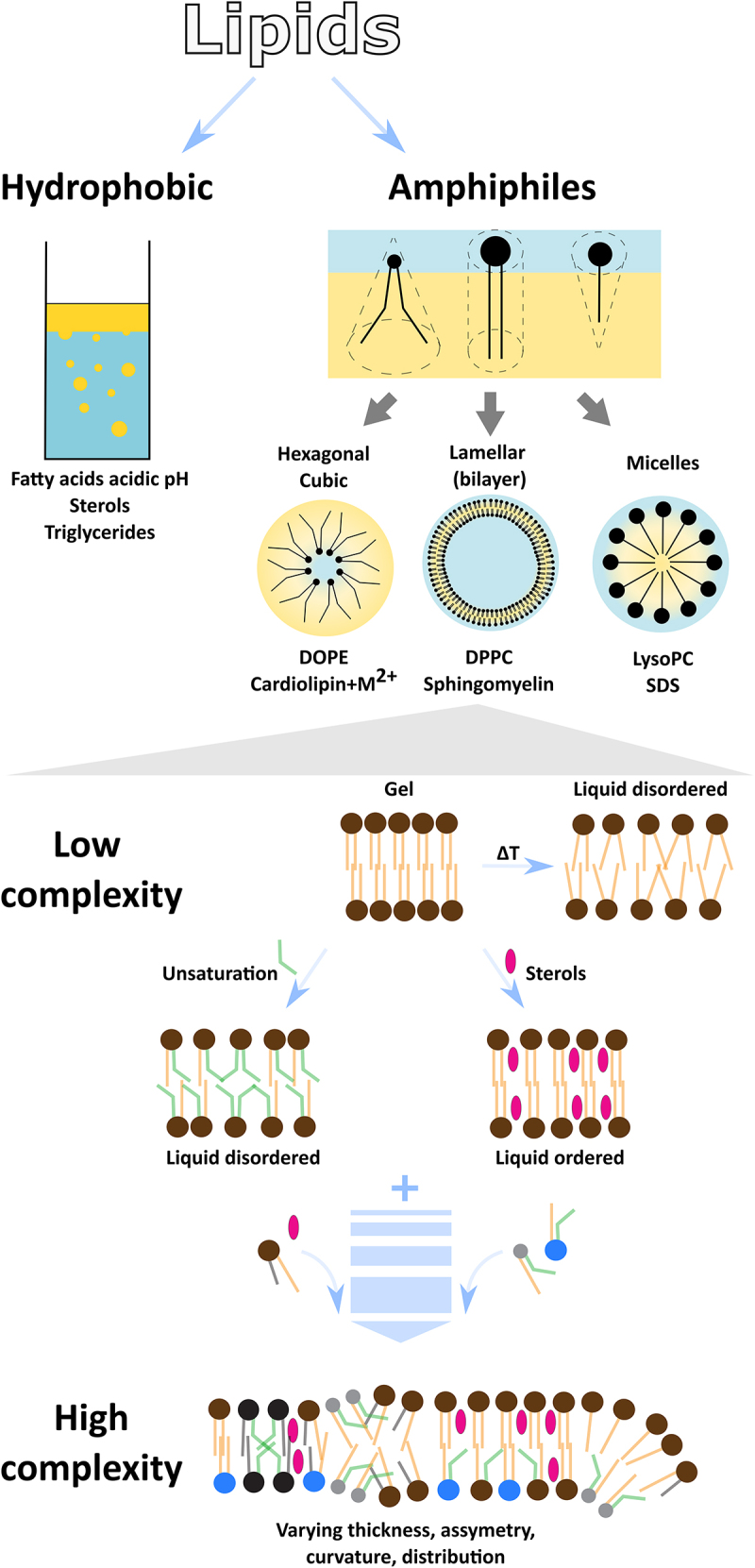
Lipids can spontaneously form a broad variety of structures. Hydrophobic compounds completely separate from water solutions whereas amphiphiles form micelles (lysolipids, detergents), inverted hexagonal or cubic phases (unsaturated phosphatidylethanolamines such as DOPE, cardiolipin in the presence of divalent ions [77]), and lipid bilayers (phospholipids such as DPPC, sphingomyelin). Lipid bilayers (membranes) can be formed just from one lipid (low complexity) which can determine the membrane’s phase. For example, a lipid membrane composed of saturated DPPC is in the gel state, however if temperature is increased (> 41 °C, ΔT), or membrane composition is altered (unsaturation of the fatty acid chains, presence of other molecules such as cholesterol) lipid membranes can form liquid phases. Further addition of other lipids with different characteristics (different shape, charge, fatty acid length and saturation, headgroup) increases the complexity which further leads to various lipid membrane phenotypes (asymmetry between leaflets, thickness, curvature).

In biological systems the most common lipids are amphiphiles with hydrophobic hydrocarbon chain(s) and hydrophilic head groups [76]. Depending on the chain-to-headgroup size ratio, different types of structures can be formed (Figure 3). For example, dioleoylphosphatidylethanolamine (DOPE) having small headgroup and long unsaturated hydrocarbon chains, has a conical shape, which cannot form a lipid bilayer itself, but an inverted hexagonal phase. Single-chained SDS detergent forms micelles in pH > 7, as well as lysophospholipids; double-chained phosphatidylcholine (PC) with its cylindrical shape can form a lipid bilayer. A broad variety of structures can be achieved when different lipid species are mixed. Indeed, in living organisms the lipidome is highly complex [78–80] and depending on the lipid distribution different phenotypes can arise [81]. For example, the local presence of a hexagonal phase has an impact on lipid membrane fusion and activity of some enzymes [82,83]. The mixture of sphingomyelin and cholesterol can form lipid rafts which have an important role in protein activity and signalling [73,84]. Furthermore, depending on the lipid composition and environment conditions, lipid membranes can have different biophysical phenotypes such as curvature, thickness, and asymmetric distribution of lipids across the two leaflets of the bilayer.

Depending on the type of the membrane lipids and temperature, the lipid membrane can be in a solid gel crystalline state (s), a liquid-disordered state (L_d_), or a liquid-ordered state (L_o_). Gel membranes are highly ordered and the lipids do not laterally diffuse, whereas liquid-disordered membranes have a high lateral lipid mobility. Liquid ordered membranes have lower lateral mobility of the lipids compared to liquid-disordered membranes; however, they are less compressed compared to gel membranes. The transition from the gel to the liquid state can occur through different mechanisms. For instance, single-component dipalmitoylphosphatidylcholine (DPPC) membrane is in the gel state at room temperature (24 °C), undergoes pre-transition above 28 °C (ripple phase with rearranged lipid molecules within the membrane [85,86]), and melts into liquid-disordered state at 41 °C. The melting temperature depends on the length of the fatty acid chains: the shorter the chains the lower the melting temperature. Furthermore, introduction of cis-double bonds in the fatty acid chains significantly decreases the melting temperature of the lipid. Additionally, when cholesterol molecules are introduced to gel-phase lipid membranes they promote lateral diffusivity while maintaining acyl-chain order, forming liquid-ordered membranes. Thus, the phase transition behaviour and lipid membrane state depend on multiple parameters, such as lipid composition (single vs multicomponent membranes), temperature, and other factors such as the presence of metal ions (for example, the presence of divalent cations can increase melting temperature of lipids) in the microenvironment of the lipid membrane [87].

In nature, biomembranes are in a liquid state, typically falling between lipid liquid ordered and disordered phases, whereas gel membranes are not generally considered to be physiologically relevant. The important feature of naturally existing lipid membranes is bilayer asymmetry [88–90]. In this case, multi-component lipid membranes do not have the same composition of lipids in the inner and outer leaflets. The asymmetry is achieved through the enzymatic activity of flippases, floppases, scramblases. However, some lipids, due to their shape, have a preference to enrich within a particular leaflet [83]. Lipid membrane asymmetry is crucial for cellular metabolism. For instance, a recent study demonstrated how genetic disruption of flippase/floppase activity has substantial effects on cellular energy flow [91].

All cells are encapsulated by a lipid bilayer that is typically built of phospholipids, sphingolipids, and sterols or hopanoids. This arrangement ensures that a cell is separated from its environment and a homoeostatic microenvironment within the cell can be maintained. Another important feature of the cell membrane is selective permeability; membranes are not a solid wall between outer and inner environments, and they are selectively permeable to some molecules, whereas other molecules need special machinery to be transported across the membrane [92]. Depending on the environmental conditions the lipid membrane can change its composition which ensures that cells will acclimate to changing environment conditions [93,94]. Lastly, lipid membranes are also important for cell metabolism by facilitating a broad range of cell signalling processes [95].

An open question is when exactly lipids started to be a vital part of living cells? Fossil records indicate that lipids derived from cells might have been present already 500–2700 million years ago [96,97]. However, due to organic matter degradation and contamination from fluid migration, the likelihood of discovering older records is low [98]. Studies suggest that simple lipids could have been synthesized on the early Earth through a variety of chemical reactions. For example, the Fischer-Tropsch reaction might be responsible for synthesis of the first amphiphilic alkanes [99,100]. There is a possibility that simple hydrocarbons, hydrocarbon alcohols, and fatty acids could have formed lipid aggregates in prebiotic scenarios [101] and the plausible presence of other molecules such as glycerolipids [102–105] and pyrene derivatives [106] might additionally stabilize these aggregates over a broader range of prebiotic conditions. Moreover, lipid species might have been synthesized outside the Earth surface and delivered in the form of meteorites [107]. There is no clear evidence that lipid-based protocells were the first type of cells which arose during evolution; however, there are multiple advantages of the lipid-based prebiotic World hypothesis, which we consider presently.

Lipid membranes can self-assemble spontaneously without a source of energy. If lipids have longer hydrocarbon chains they can spontaneously form aggregates at lower concentrations. It is possible that, during evaporation and rehydration, freeze-thawing cycles, temperature cycling, or accumulating on the air–water interfaces lipid aggregates can bind or encapsulate different types of molecules [34,108–111]. The encapsulation process might have an evolutionary advantage: encapsulated molecules can be protected from the external lipid vesicle environment. Additionally, encapsulated microenvironments can be different compared to the outer environment, with different concentration of solutes, molecular crowding, and macromolecule composition, which provides one possible path to the creation of the first protocells [102,112–116]. Finally, lipid membranes might act as an organizational scaffold for different types of molecules. Scaffolding would have several advantages in the prebiotic world: not only would different types of molecules be in close proximity on the membrane surfaces, but also the membrane might act as a chaperone [112], potentially contributing to higher biomolecular stability. Additionally, lipid membrane lattices might have a templating activity, serving a role in the synthesis of polymers [32,33] on dynamic fluid membranes rather than solid mineral surfaces [51].

Thus, lipids could potentially act as a molecular organization platform in prebiotic scenarios. In the RNA World hypothesis, the presence of lipids might have greatly contributed to the emergence of the first biochemical networks: RNA with the help of lipids might have created robust and responsive platforms which further evolved into the first protocells. To further develop the idea of the RNA–lipid World hypothesis we need to understand if interactions between those two molecules is possible, and what would be the affinity and binding mechanisms of RNA–lipid complexes.

## 5. Mechanisms of RNA–lipid interactions

One of the first indications of RNA–lipid interactions was described in 1978 [7,117]. It was observed that RNA binds to lipid membranes in the presence of divalent cations, which causes aggregation of lipid vesicles. The binding process was reversible: the addition of EDTA, heparin or monovalent salts significantly decreased RNA–lipid interactions, suggesting a purely electrostatic type of interaction. It was suggested that nucleic acids can be partially unfolded by lipid membranes, and the presence of a lipid membrane can partially protect RNA from being digested by RNases. Those observations became a foundation for future research (briefly summarized in Table 1) aimed at understanding the features of RNA–lipid interactions, specifically: what is the morphology of RNA–lipid complexes, what is the effect of the binding on RNA structure and stability, and what environmental conditions facilitate binding? A broad variety of literature also includes descriptions of DNA–lipid interactions, which can also partially describe the nature of RNA–lipid interactions due to the similar nature of both RNA and DNA species.

**Table 1.** A short list of methods that have been used to determine the interaction between nucleic acids (RNA, DNA) and lipid membranes.

Method	Experimental setup	Main results	References
Langmuir monolayer isotherms, Brewster angle microscopy (BAM)	Amphiphilic lipids spread on the air-water interface creating a monolayer (hydrophobic parts face air, hydrophilic - water). Lipids can be compressed (from extended to gel-like phases) which changes the surface tension and monolayer phenotype (which can be detected using BAM). The presence of the binding partners (such as RNA or DNA), ions, and changes in temperature can change the organization and compression of lipids. This method gives insights into lipid monolayer organization and dimensions (for instance area per lipid headgroup), as well as binding affinities.	Nucleic acids interact with lipid surfaces in the presence of divalent ions. Interactions depend on the lipid packing with partial penetration of the lipid monolayer with lower lipid packing and exclusion of nucleic acids from the monolayer with high lipid packing. Lipid-associated nucleic acids complex lipids, change membrane packing, and change surface potential. There is different binding of double stranded and single stranded nucleic acids to differently packed lipid membranes.	[14,121,123–125,128]
Infrared (IR) spectroscopy	IR waves are absorbed by different covalent bonds at various vibrational states, providing information about lipid membrane composition, phase state, and the binding of other molecules (e.g. shifts or changes in the intensity of absorption bands).	DNA interacts with the lipid membrane in the presence of divalent ions which does not disrupt order of lipid chains. DNA might interact with the hydrophobic part of the lipid membrane.	[121,124,128,137,139]
Circular dichroism (CD)	RNA molecules absorb left or right-hand circularly polarised UV light differently which gives a differential spectrum. The shape of the CD spectrum gives insights into RNA structure (single stranded, double stranded, G-quadruplexes) and structural changes (melting, structural transitions).	RNA is in the A-helix form while interacting with lipid membranes. Negatively charged (in Suga et al. neutral membranes as well) lipid membranes decrease RNA stability, while positively charged membranes increase RNA melting temperatures.	[8,137–139]
X-ray and neutron scattering	X-rays or neutrons are reflected from lipid bilayers giving information about lipid membrane thickness, homogeneity (phase separation), and binding of membrane interaction partners.	DNA interaction with the lipid bilayer introduces sorting of metal ions and water molecules (partial dehydration of the lipid membrane). Metal cations bound to one phospholipid bridge the interaction with DNA by exposing the positive charge of the lipid headgroup. Alternatively, metal ions create a salt bridge between lipid and nucleic acid phosphates.	[14,119–122,124,125,128]
Atomic force microscopy (AFM)	AFM tips (2–15 nm) tap the surface of RNAs present on the lipid bilayer. This method determines the size, shape, and aggregation of RNAs.	RNA binds to lipid membranes resulting in different different “phenotypes” depending on lipid composition. Lipid binding RNAs form macromolecular RNA-RNA complexes. Complexes prefer to bind to the imperfections in flat lipid membranes (edges).	[137,151]
Quartz crystal microbalance	An oscillating quartz sensor detects changes in the resonance frequency which is altered by RNA–lipid binding events.	tRNA associates with lipid liquid and gel membranes. Lipid membranes can serve as a template for RNA-RNA interactions.	[13,140]
Differential scanning calorimetry (DSC)	Changes in the heat capacity (caused by, for instance, lipid-nucleic acid interactions) can be detected by a calorimeter. These changes give a hint on how nucleic acids interact with different lipid membranes, or how the binding changes the melting transition of lipid membranes.	RNA influences the lipid membrane phase transition.	[13,159]
Fluorescence spectroscopy	Lipid membranes are stained with lipophilic fluorescent dyes in the presence of specific RNAs and divalent ions. Changes in fluorescence intensity and/or emission spectrum shifts determine changes in lipid membrane properties. Alternatively, nucleic acids are stained with base intercalators to measure stability of nucleic acid folding.	RNA influences lipid membrane phase transition and lipid ordering. DNA is stabilised in the presence of lipid membranes against thermal denaturation (the effect is dependent on the type of cations present). Plausible incorporation into the lipid membrane.	[8,12,127,139]
UV absorbance, dynamic light scattering (DLS)	RNA absorbs light in the UV range. Changes in absorbance might indicate RNA base pairing. The DLS method is based on detection of light scattered by different molecules. The readout depends on, among other factors,the size of monitored particles. Note: UV-based crosslinking can also be utilized to determine the effect of lipid species on RNA-RNA interactions.	RNA and DNA causes superior lipid vesicle aggregation which is reversible upon addition to divalent cation chelators (EDTA, EGTA). The precise phenotype of aggregates is not known. RNA–lipid binding can increase the melting temperature of nucleic acids. Purines (A and G) bind better to the fatty-acid based micelles compared with C and U.	[5,8,127,133,137,139,142]
Ultrasonic spectroscopy	Ultrasonic waves are disrupted in different ways (changes in velocity, attenuation) while passing through the lipid membranes of different composition and phase. Binding can change the readout.	The DNA-lipid binding ratio is estimated to be 5 lipids:nucleotide. Changes in the melting phenotype of lipid membrane. The DNA used was not a defined sequence but rather a natural extract of calf thymus DNA.	[118]
Fluorescence microscopy	Fluorescently labelled lipid membranes and nucleic acids are visualised under the fluorescent or confocal microscope. Co-localisation of RNA and lipids can be directly observed (signal distribution, FRET).	RNA/DNA is in close proximity to lipid membranes, with preference of more ordered membranes. Reversible binding upon phase separation -> melting cycles.	[5,12,130,140,151]
Size exclusion chromatography (SEC)	Chromatography resin separates molecules based on their size. RNAs associated with large lipid aggregates elute differently compared with free RNAs. Lipid-binding RNAs can be collected, amplified, and used for another SEC. After several selection cycles the enriched pool of RNAs is collected and sequenced. The experiment can start from a random pool of RNAs and end with specific lipid-binding RNAs.	Multiple lipid-binding RNAs were identified. The lipid-binding RNAs usually form large RNA-RNA complexes. So far no cross-selectivity (for instance towards lipid membranes composed of different lipids or of different fluidity) was evaluated. RNA has higher affinity towards ordered lipid membranes compared with lipid liquid membranes. Highest binding observed for lipid gel membranes.	[10–12,150,160,161]
Centrifugation	Lipid membranes are spun down at high centrifugal forces (>90k xG for small lipid vesicles). RNAs which are bound to the vesicles co-migrate with liposomes to the pellet. Both supernatant and pellet can be analysed (radioactivity, gel electrophoresis, absorbance, Qubit) to determine bound and unbound RNA fractions. Limitation: RNA–lipid aggregates (lipid gel membranes) sediment faster compared to liquid membranes, thus centrifugation times and separation quality might differ.	RNA interacts with lipid gel membranes, whereas binding to lipid liquid membranes is limited. Calculated lipid-buffer partition coefficients.	[5–7,117]
Magnetic bead-based binding assay	Liposomes contain a small amount of biotinylated lipid which is binding to streptavidin-coated magnetic beads. Lipid-binding RNA can be separated from the buffer solution using a magnetic field. Non-bound RNA can be quantified in order to calculate binding partition coefficients. Note: method can be also potentially utilised for *in vitro* selection instead of SEC. However, the method is dependent on the lipid-bead coupling (for example streptavidin-biotin), and precise concentration of available lipid species (exposed on the bead) has to be measured; tight packing of lipids on bead surfaces can potentially bias the experiment.	RNA interacts with lipid gel membranes, whereas binding to lipid liquid membranes is limited. Calculated lipid-buffer partition coefficients.	[5]
Single particle profiler (SPP)	Confocal-microscopy based setup coupled with fluorescence correlation spectroscopy (FCS). Fluorescently labelled lipid particles bind to the fluorescently labelled RNA species. Multi-channel detection allows determination of binding/encapsulation efficiency, as well as the fluidity of the lipid membrane.	mRNA encapsulation efficiency and heterogeneity evaluated with high precision (single particle resolution).	[162]
Calculations, molecular dynamics (MD) simulations	Mathematical model-based simulations of short nucleic acids (fixed sequences or structures) with simple defined lipid membranes. Limitation: force fields used for nucleic acids simplify the lipid membrane and vice versa. Introduction of divalent ions into the same RNA–lipid simulation is not straightforward.	DNA interacts with the zwitterionic lipid membranes in the presence of divalent ions. RNA interacts with lipid membranes via hydrogen bonding, with a strong preference for guanine.	[126,129,130,134,156–158]

Note that for binding assays, multiple approaches can be used to separate lipid-bound from unbound RNAs. We present only a few selected methods here; however, any technique that allows physical separation and quantification of RNA (e.g. native polyacrylamide gel electrophoresis, affinity chromatography, ultrafiltration) would be sufficient to calculate binding partition coefficients. It is also important to consider the source of the DNAs/RNAs used. In some cases, bulk or generic nucleic acids (e.g. calf thymus or sperm DNA, tRNA) were used. However, in certain studies, specific RNAs (e.g. RNA9:10 or other sequence-defined molecules) were used, which can significantly influence the results.

### 5.1. Nucleic acid-lipid binding is dependent on ion composition

**Figure 4.**
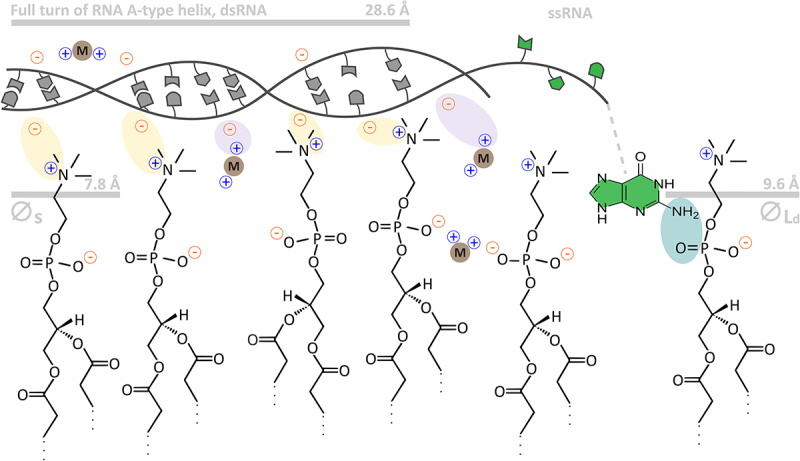
Schematic representation of RNA – lipid interaction. Double-stranded RNA interacts with lipid gel membranes (left side of the picture) through electrostatic interactions between its negatively charged backbone and either positively charged choline residues (yellow spheres) or metal ions (light purple spheres). Lipid gel membranes are tightly packed (around 48 Å^2^ per headgroup for DPPC [131]), allowing the RNA to interact only with their surfaces (the average diameter of the PC headgroup is 7.8 Å). For comparison, one full twist of an A-type RNA helix is around 28.6 Å (the distance between minor grooves of the helix, in which the negatively charged phosphate backbone faces the lipid membrane surface). Nucleic acid bases (simplified here as dark-grey shapes inside the RNA helix) do not have access to the lipid membrane surfaces. On the other hand, lipid liquid membranes (around 72 Å^2^ per headgroup for DOPC [132]; in this case, the diameter of the headgroup is larger than in gel membranes) can be slightly penetrated by single-stranded RNA (ssRNA, right part of the picture), allowing some nucleic acid bases (simplified here as dark-green shapes and represented by guanine) to interact with the lipid headgroup area through hydrogen bonding (dark green circle). The scheme is a simplified representation; thus, the ratio of binding partners is not 100% accurate. Longer fatty acid chains (at the bottom of the scheme), which form the hydrophobic core of the lipid membrane, are not shown to simplify the picture.

Several studies focused on what ion compositions facilitate nucleic acid – lipid membrane binding. Divalent ions, especially calcium and magnesium are essential [7,10,117–130]. It is, however, not entirely clear how the presence of those ions can facilitate binding. Two principal models have been proposed (Figure 4).

The first model of nucleic acid – metal ion – lipid membrane interactions states that divalent ions directly contribute to interactions between the negatively charged nucleic acid backbone and the phosphate group from the phosphatidylcholine species. It was suggested that the binding of divalent ions to lipid membranes changes the net charge of the membrane to positive, which attracts the negatively charged nucleic acid backbone, eventually leading to the divalent ion driven nucleic acid-membrane binding [7,117,120,129,130,133].

The second model states that divalent ions bind to the negatively charged phosphate part of the phospholipid, which exposes a positively charged choline group. This model of action assumes that ions can penetrate the headgroup area and bind to the negatively charged part of phospholipids. Indeed, it was shown both *in vitro* and *in silico* that divalent ions can interact with phospholipids leading to changes in phospholipid geometry: for example, the choline group of phospholipid is straightened and tilted towards water [134,135], which in principle might lead to the changes in membrane net charge from neutral to cationic [119,121,124,126], which further supports nucleic acid – lipid membrane interactions.

There is a preference for particular divalent ions in nucleic acid – phospholipid interactions. McLoughlin et al. determined that binding of DNA to the zwitterionic phospholipid membrane is enhanced by divalent ions to relative degrees in the order of Ca^2+^ > Mg^2+^ > Ba^2+^. This is perhaps because of a particular ion’s radius being preferred by the lipid and nucleic acid phosphate backbones [123,134,136]. In another study, different transition metals were checked in terms of inducing DNA-DOPC binding and the Cu^2+^ > Ni^2+^ > Co^2+^ > > Ca^2+^ > Mg^2+^ = Zn^2+^ order was determined [127].

Another ion-based factor which can modify nucleic acid-lipid binding is the presence of monovalent salts. It was described that higher concentrations of NaCl can reduce nucleic acid-lipid binding in divalent ions containing buffers [7,8,12,117,124]. The aforementioned effect is most likely based on competition: monovalent salts binding to the lipid membrane and to the nucleic acid backbone excludes divalent ions. Monovalent ions can’t bridge the electrostatic interaction between nucleic acid and lipid backbones, thus decreasing lipid binding.

In most cases, divalent ion driven nucleic acid binding leads to liposome-aggregation, which is fully reversible by the addition of a metal chelator, for example EDTA [5,7,8,117]. In particular, lipid gel membrane vesicles are most prone to aggregation by nucleic acids, possibly because they exhibit the strongest affinity for nucleic acids [5,12]. Melting the membrane into its liquid phase leads to disaggregation. At the molecular level, it was suggested that lipid membranes aggregate due to interaction of several vesicles with one nucleic acid molecule, where the nucleic acid is ‘sandwiched’ between two or more lipid vesicles [120]. Alternatively, it is also possible that vesicles are aggregated by one long RNA molecule, since it was also described that heparin (a long negatively charged sugar polymer) can bind to lipid vesicles [7,117].

### 5.2. Nucleobases interact with the lipid bilayer in close proximity to the hydrophobic core

Despite the highly hydrophilic character of nucleic acid molecules, the presence of nucleic acid bases in close proximity to the hydrophobic core of lipid membranes has been observed. Using infrared spectroscopy, Marty et al. observed that guanine, adenine, and uracil can interact with lipid aliphatic chains [137]. Using a similar methodology, Suga et al. confirmed nucleobase – aliphatic chain interactions, which additionally led to dehydration of RNA phosphate backbone and conformational change (destacking of nucleic acid bases) of tRNA species [138,139]. Similar observations were reported by Michanek et al., where single stranded RNA species were able to interact with hydrophobic hydrocarbon chains of phospholipids, whereas no such effect was visible for double-stranded nucleic acid species [13,14,140]. Such observations suggest that unfolded single stranded RNA species have exposed nucleic acid bases that are capable of interacting with the deeper regions of phospholipid membranes, whereas such a mechanism is not possible for double stranded species, where nucleobases are buried within RNA/DNA helices. For higher lipid packing no such interaction was observed, which suggests that RNA is expelled from ordered gel lipid domains [14,121,124] due to the restricted space between lipids (change of the phase from liquid to gel). In synthetic systems, lipid membrane penetration by nucleic acids can be artificially induced via hydrophobic anchors present on the nucleic acid [130,141].

Notably, nucleic acid bases, especially purines, can interact with fatty acid-based membranes, and one of the suggested forms of interaction involves the insertion of nucleic acid bases into the membrane [142]. Such interactions between aliphatic hydrocarbon chains and RNAs might be also responsible for disrupting and increasing the permeability of lipid membranes – it was reported that RNA selected against liposome targets through systematic evolution of ligands by exponential enrichment (SELEX) increases membrane permeability for the small solutes and can disrupt membrane integrity [10,11,143], and selectively increase the permeability for co-bound amino acids [143]. It is worth noting that, in a series of *in vitro* selection experiments, a set of RNAs were found which are specifically binding to the hydrophobic amino acids such as valine [144], tryptophan [42,145], phenylalanine [146], and isoleucine [147–149]. Several RNA structures were characterized and described as hydrophobic-moiety binding pockets, which confirms that, in principle, it is possible that RNA species can bind to hydrophobic aliphatic chains of lipids.

### 5.3. Lipid membrane and nucleic acid properties determine binding affinity

As with all of the interactions present in molecular biology, RNA–lipid interactions are strictly dependent on the features of both of the binding partners. In general, it has been reported that RNA–lipid binding is dependent on lipid membrane fluidity, composition, and RNA sequence and structure.

A number of *in vitro* selection experiments have shown that only a narrow pool of RNA molecules can bind to liquid lipid membranes. In the early 2000’s, the group of Michael Yarus characterized RNA molecules which can bind liquid lipid membranes, which also influences membrane permeability [10,11,150]. Studies have shown that RNA species binding to lipid membranes likely form intermolecular macro-complexes. In some cases, binding assays on individual sequences exhibited little to no lipid-binding activity [11,150,151]. In the following years, it was reported that, despite high RNA sequence and structure selectivity required for binding to liquid membranes, RNA binding to ordered and gel membranes is less dependent on RNA features and even a random mix of RNA species binds to the gel membranes with significantly higher affinity compared to liquid membranes [5,6,8,12]; gel membrane preference was also observed for a DNA [130] and phosphorothioate [152] species. Interestingly, the opposite effect was also described: Michanek et al. reported that higher adsorption of tRNA species was observed for liquid disordered compared to gel-phase membranes [13]. A few approaches were conducted in order to measure nucleic acid-lipid interaction binding affinity: Khvorova et al. reported a dissociation constant of 1x10^−12^ to 1x10^−13^ M for RNAs and PC:cholesterol-based membranes, whereas Lu and Rhodes determined dissociation constants in the range of 550–750 nM for phosphorothioate oligomers and gel membrane DPPC vesicles [10,152].

Another feature which directly changes nucleic acid-lipid binding is lipid composition, i.e. type of the lipid headgroup. Lipid headgroups with positive net-charge naturally attract negatively charged nucleic acid species – this feature is used in nucleic acid-based drug formulations [9]. Zwitterionic lipid headgroups bind RNA species with different affinities: depending on the buffer composition and membrane fluidity different binding can be observed (see above). Surprisingly, despite a negative net charge, RNA binding to the negatively charged lipid membranes was also observed; however, this binding mechanism remains unknown [8]. Interestingly, the binding of nucleic acid species to lipid membranes can also change the characteristics of the membrane in which dehydration [122], increased membrane permeability [10,11], and altered membrane melting behaviour [8,12,13] were reported.

RNA sequence has a vital role in RNA-liquid membrane binding most likely due to structural effects: particular RNA sequences form tertiary structures which bind to the lipid membrane. It is, however, not clear if sequence per-se has an influence on membrane binding, i.e. if particular nucleobases have preference for the lipid membrane. Analyzing hydrophobicity indexes [153,154] showed that RNAs composed of the most hydrophobic nucleobase, adenine, should bind to lipid membranes with highest affinity, however few reports confirm this hypothesis [142,155]. Khvorova et al. described that liquid membrane binding RNA sequences have increased guanine content; indeed, several guanines were protected from RNase T1 digestion during liposome binding, however it was not clear if this is lipid-based screening or structural RNA changes [10]. Lu and Rhodes reported that purine-rich phosphorothioate oligonucleotides bind with higher affinity to lipid membranes [152]. Based on the data, purine-based oligonucleotides bind to the lipid membranes with higher affinity, however the mechanism is unknown. Currently, the importance of guanine as well as the structure of RNA on the RNA–lipid interaction was demonstrated in the case of lipid gel membranes [5]. Recently it was further shown *in silico* that the RNA–lipid interactions indeed depend on the membrane fluidity, and RNA sequence; in particular, in agreement with experimental data, the RNA–lipid gel membrane interaction is stronger compared with lipid liquid membranes and that the presence of guanine can enhance RNA–lipid binding via hydrogen bonds between the nucleobases and lipid headgroup area [156–158].

Depending on the type of membrane, methodology and experimental conditions, different lipid to nucleic acid-binding ratio values have been reported. Using temperature-scanning ultrasonic study and X-ray diffraction, the binding ratio between calf thymus DNA (ctDNA) and DPPC or DOPC membranes ranges between 4.5–5 lipids per nucleotide in the presence of the Ca ions [118,122]. Ratios of 1:1 and 2:1 lipid:nucleotide were also determined [121,126]. For the liquid membranes (or, to be more precise, liquid extended monolayers) a ratio of 7.5 lipid:nt was described [14]. A similar range of RNA–lipid binding was reported for lipid gel membranes in which the correlation between the binding and RNA:lipid ratio was shown with ‘strong’ binding (double stranded RNA) being represented by 1:1 lipid-to-nucleotide ratio and ‘weaker’ (single stranded RNA) a by larger amount of lipid per nucleotide [5].

All in all, RNA–lipid binding depends on several factors that play a crucial role in determining binding affinity. Lipid membrane fluidity, net charge and composition, as well as RNA sequence and structure, are all key factors that can influence the affinities of RNA–lipid interactions.

### 5.4. Influence of lipid binding on RNA activity

Despite the growing number of studies examining RNA–lipid interactions, not much is known about the influence of lipids on RNA activity. Anella et al. reported that the presence of fatty acid aggregates influences the activity of L1 ligase, however the precise mechanism was not determined. It was hypothesized that higher concentrations of fatty-acid-based structures uptake divalent ions from the solution, which further leads to decreased catalytic RNA activity [163,164]. By using a cholesterol-modified RNA substrate, Müller and Bartel improved the efficiency of the RNA polymerase ribozyme, most likely via increasing local concentrations of the reactants [165]. Suga and colleagues showed that the presence of lipid membranes might influence RNA conformation and self-cleavage activity [138,139,166]. Moreover, RNA base-pairing can be also influenced by lipid membranes [5,8,140]. Recently, we have shown that the presence of lipid membranes can influence the activity of R3C ligase [5], HDV, and hammerhead [6] ribozymes. Namely, lipid membranes can both inhibit and increase the reaction progress of different ribozymes and these effects are dependent on the sequence of RNA as well as on the lipid membrane composition. Lastly, the presence of lipid liquid membranes can influence RNA integrity with higher RNA degradation rates for lipid liquid membranes [6]. RNA encapsulation has also been shown to influence RNA phenotypes as well [105,112,113,115], which introduces lipid membranes as a flexible and complex platform for controlling RNA activity. Lipid-dependent control of RNA occurs not only through direct RNA–lipid interactions but also by altering the RNAs microenvironment, similar to coacervate-based systems [167].

## 6. Life in an RNA–lipid world

What would be the plausible evolutionary advantages of RNA–lipid interactions for early life, and what might these interactions add to the so-called RNA World Hypothesis? To understand that, we must hypothesize about what challenges RNA-based systems could face in a prebiotic World.

One of the most obvious problems in the prebiotic World is absence of any sophisticated and evolved regulatory mechanisms, which are present in modern biology. Lack of control and spatial distribution of catalytically active RNA molecules might have resulted in rapid chemically driven RNA degradation. The presence of lipid membranes might have provided a flexible scaffold and primitive organization platform, which could regulate the activity and coordinate the localization of RNA molecules. Additionally, non-RNA membrane-enriched molecules such as metal ions might have improved the general stability and activity of RNA.

It is likely that the concentration of RNA in the prebiotic world was very low due to the low synthesis rates and large volumes of prebiotic water reservoirs. It might have been possible, however, to increase the concentration of molecules via evaporation or freezing processes [39,168]. It is also possible that minerals acted as an RNA scaffold [169,170], theoretically increasing local RNA concentrations. However, the stiff nature of mineral surfaces could also be a disadvantage: RNA species present on surfaces might be trapped and, furthermore, have no chances to interact with other RNA molecules. Lipid membranes, however, can undergo phase transitions upon temperature changes, resulting in the introduction of reversible RNA binding mechanisms.

Another advantage of RNA–lipid interactions in a prebiotic World is the introduction of a microenvironment with significantly different physical and chemical properties. Self-assembled lipid membranes that bind RNA molecules, would lead to increased local concentrations and reduced dimensionality of the system. Additionally, close proximity of the hydrophobic core with lower dielectric point and water activity might be another factor that, in general, might change RNA fitness, stability, and introduce additional factors in the process of evolution.

Moreover, the presence of lipid membranes might catalyse RNA-chain elongation [32,33]. Rajamani et al. have described that chains as long as 100 nucleotides can be synthesized in a system based on lipid membranes, which means that lipid membranes might have acted as primitive RNA-biofactories promoting polymerization in the prebiotic World.

**Figure 5.**
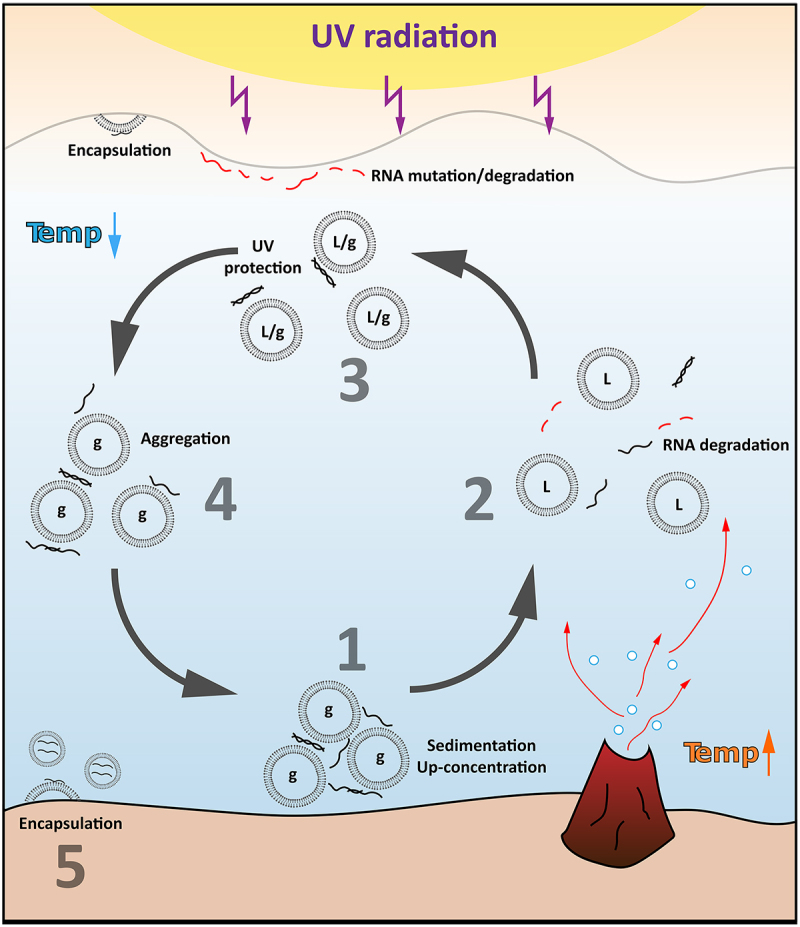
RNA – lipid prebiotic scenarios. 1. RNA binds to lipid gel (G) membrane vesicles, leading to the up-concentration of RNA and sedimentation in primordial aqueous environments. 2. A thermal vent locally increases the temperature and induces flow, which brings RNA – lipid aggregates to the surface and melts lipid membranes (L), triggering RNA degradation. Only the fittest RNA can survive. 3. At the surface, the temperature drops and lipids undergo a liquid-to-gel phase transition. RNA bound to lipid vesicles may be protected from surface UV radiation; RNA and lipids can accumulate at the water – air interface, leading to plausible RNA encapsulation. 4. Lipids are in the gel phase, which increases the amount of bound RNA. RNA – lipid complexes sediment again. 5. Mineral-surface-based budding lipid membranes encapsulate evolved RNAs, creating a pool of diverse protocells that undergo further development. Catalytically active RNAs can generate a new pool of biomolecules and metabolites, sustaining a homoeostatic environment.

One can imagine that prebiotic conditions (hydrothermal vents, freeze-drying cycles, temperature shifts, UV radiation) would introduce the system in which, for example, lipid membranes undergo a liquid-gel phase transition, which will further affect RNA–lipid binding and RNA stability. Binding-cleavage cycles would then provide a basis for evolution in which stable and longer and structured RNAs would have larger chances to enrich. Additionally, in aqueous environments, RNA–lipid aggregates would preferentially sediment to the bottom of the water column which would then protect RNAs from the surface UV radiation, up-concentrate RNA species (increasing the chances of RNA–RNA interactions and trans-ribozymal activities), and expose the system to an environment with different features (temperature and chemical gradients, hydrothermal vents). After the creation of the first RNA–lipid-based protocells the evolution process can start with selection of RNAs being more active, stable, and responsive to the changes of the local encapsulated environment as well as outer conditions (Figure 5). Finally, the primitive metabolism could be also established in which RNA, theoretically, can utilize its aptamer and catalytic activity to introduce rTCA [44]. It was shown that the presence of citrate, one of the components of TCA, improves the fitness of RNA–lipid-based systems [171] which potentially could play a role in selective recognition and compartmentalization of citrate which could have facilitated the evolution of TCA-centric metabolisms [172].

The RNA–lipid interplay can, in principle, mimic modern protein-based systems. For example, the degradation of RNA in the presence of certain lipid species is analogous to the activity of RNase E [173], which is a lipid-membrane associated enzyme. The differential RNA–lipid binding (single stranded vs double stranded RNA), as well as RNA folding and unfolding is similar to the activity of G3BP protein which recognizes unfolded RNAs and creates macromolecular RNA-protein complexes [174]. Changing RNA catalytic activity through the interaction with lipid membranes resembles protein-based systems in which the interaction with binding partners can inhibit enzymatic activity in the Michaelis-Menten-like kinetics (for instance, allosteric inhibition). Moreover, selective RNA–lipid binding might act as a lipid composition and a fluidity biosensor, similarly to the Mga2 protein [75]. The viral assembly of Human Immunodeficiency Virus (HIV) includes a step in which Gag protein sorts lipids and binds specifically to phosphatidylinositol (PIP [175,176]) – this specific lipid binding can be also achieved purely by RNA [177], which opens the speculation about protein-free proto-virus assembly. Lastly, increased probability of RNA-RNA interactions in the presence of lipid membranes would act as a prebiotic RISC system [178], which modern cells use as a protein based-machinery to recognize and degrade parasitic RNAs.

Merging all of those possibilities, the RNA–lipid World is an attractive problem to hypothesize about [15,179,180] and experimentally develop. Lipid-based RNA synthesis, which might have evolved into a robust and selective production of different RNA species, is analogous to the modern protein-based World, where changes in the environment might’ve led to changes in general RNA composition in the system. It is plausible that ancient RNA–lipid interactions provided a tunable system based only on two biomolecules.

## 7. RNA–lipid interactions in synthetic biology

One of the principal aims of synthetic biology is to mimic or synthesize life through a bottom-up approach. Simply put, the bottom-up approach uses simple building blocks such as proteins, nucleic acids, lipids, and other molecules to form complex, responsive and robust life-like systems, which eventually can be engineered into an artificial cell [181]. The main problem of synthetic systems is the relatively high complexity required in order to achieve complex functions that are robust. For example, in order to achieve a vesicular system in which particular functional proteins must be synthesized, not only are lipids required, but also a DNA template, transcription and translation apparatus. This ‘simple’ system might’ve further required vital posttranslational modifications and protein chaperones, which further can modulate protein functionality and stability. It is, however, possible to decrease the complexity of the system by using RNA-based systems composed of ribozymes, aptamers and aptazymes [182–184]. It was shown that simple systems based on encapsulated RNA species can be functional. For example, hammerhead ribozyme activity can be maintained in lipid vesicles [102]. The main problem of facilitating ribozyme reactions within lipid vesicles is membrane impermeability for potential ribozyme substrates or metal ions delivered from the outer environment [179,185]. However, it was possible to overcome this problem by changing lipid membrane composition: using membranes composed of fatty acids and fatty acid glycerol esters, Mg ions were able to penetrate lipid vesicles, which lead to enhancement of hammerhead ribozyme activity [102]. Similarly, Adamala et al. showed that hammerhead ribozyme activity is preserved inside the lipid vesicles, however reaction rates were significantly slower compared to non-encapsulated systems [105]. Moreover, it is possible to use lipid-interacting RNA species to form molecule-specific trans-membrane shuttles. Janas et al. have shown that merging an *in vitro* selected lipid-binding RNA system (RNA9 and RNA10) with a tryptophan aptamer results in tryptophan-specific transport through the lipid membrane [143]. Lately, a similar approach was conducted with the arginine aptamer proving the universal use and flexibility of lipid-binding RNAs [161].

It is possible to artificially colocalize RNA and lipid membranes using positively charged species, such as cationic lipids [186], peptides [187], hydrophobic modifications [130,165,188], and biotin (biotinylated lipid – biotin aptamer pair [189]). However, ideally, RNA should directly and selectively bind to the lipid membranes of different characteristics (physical properties or chemical composition) which would provide a basis for responsiveness and selective compartmentalization: lipid membrane properties could be modulated by RNA and, conversely, RNA activity and structure can be tuned by the lipid membranes. It is tempting to hypothesize that RNAs could completely substitute for protein-based systems, at least in simple synthetic systems. The possibility of RNAs interacting with the hydrophobic core of the lipid membrane lead to the attractive hypothesis that RNAs can create trans-membrane channels [141,190] with activity similar to aquaporins, ion channels and, inevitably, to the ATPases generating electrochemical gradients across the membrane which might have significant implications both in synthetic biology and RNA–lipid World hypothesis. It was reported that artificial ribozymes could be developed for other chemical reactions such as carbon–carbon bond formation [191–193] and redox reactions [194–197] which practically proves that RNA-based systems can be self-sufficient without the presence of protein machinery. Merging the lipid binding and catalytic activities can, thus, theoretically lead to the development of aptazymes which can modify lipid chemistry by changing acyl chain unsaturation, cleaving and rearranging the lipid headgroup area, and, lastly, the synthesis of lipids *de novo* through RNA-based biochemistry. By modifying the lipid composition, we could further tune RNA activity and stability, leading to simple feedback systems in which RNA would act as a lipid membrane biosensor: changes in lipid membrane features would, for example, activate ribozymes, increase RNA stability, or make mRNA species translationally active.

In some cases, lipid vesicles act as a simple RNA-encapsulation platform. It was recently shown that ribozyme encapsulation promotes faster evolution, as well as stabilizes folding of nucleic acid species compared with non-encapsulated systems [113,114,198]. Saha et al. showed that encapsulated malachite green aptamer folds with a higher efficiency compared to bulk-dissolved species, which indicates that lipid vesicles might act as a chaperone for RNA species [112]. Strikingly, using RNA-origami technology, it was shown that RNA complexes can be synthesized inside lipid vesicles and co-localize with the lipid membrane using biotin–biotin aptamer pairing, which creates the amazing opportunity to use RNAs as a membrane-associated cytoskeleton which, eventually, could lead to creation of protein-free artificial cells [189].

Thus, it is possible to create simple RNA–lipid based functional systems. Further development of new ribozymes, riboswitches, and aptamers opens an exciting chemical space to engineer RNA–lipid-based artificial cells with RNA acting as catalysts, biosensors, and as an information carrier.

## 8. What’s the physiological relevance?

During the evolutionary transition from an RNA World to a protein and DNA-based world RNA–lipid interactions might have become less relevant. This shift is likely due to the short lifespan of RNA both inside and outside cells, as well as the increasing role of proteins as signalling and structural membrane-associated biomolecules. However, during the last years, there is more evidence that some remnants of RNA–lipid interactions might still be present in living cells and have functional implications.

One recent study shows that some mRNA species colocalize with lipid membranes during Drosophila embryo development. Using FISH labelling, Jambor et al. determined that mRNA coding transmembrane calcium channels (named fwe mRNA) colocalize in particular cortical domains of the ooplasts, close to the membrane surface. From the same research, Shroom mRNAs, which translate to proteins important for morphogenesis of epithelial cells, colocalize with the membranes of the epithelial cells [199]. Lécuyer et al. found out that 0.3% of mRNAs are associated with lipid membranes during Drosophila embryogenesis [200]. Another indication of mRNA colocalization with lipid species were found recently by Janas et al., where VegT mRNA colocalizes with the mitochondrial clouds of Xenopus as well as with ordered lipid rafts [201]. Colocalization of tRNA^Sec^ to the lipid membranes was also reported, however membrane binding was mostly due to the hydrophobic character of the tRNA modification [188]. Additionally, it was shown that long non-coding RNAs (lncRNAs) interact with PIP3 (a signalling membrane phospholipid), phosphatidic acid, and lipid droplets, which might have further implications in cancer development and cell metabolism [177,202,203]. Additionally, it was found that RNA species can bind to bacterial lipid membranes, with implications for protein translation and translocation [204–207]. Moreover, extracted cellular RNAs demonstrate affinity towards the artificial lipid membranes as well [208]. There is evidence that RNA can be displayed on the surfaces of cells (maxRNAs) however the function of such a phenotype is yet unknown [209]. Additionally, some RNA species might be involved in exosome formation, and be preferentially packed inside the exosome lumen [160,210–215]. It was shown that nuclear sphingomyelin might interact with dsRNA species, which protect RNA from RNase-based degradation [216]. Lastly, it was recently shown that novel RNA species, glycoRNAs, are associated with cellular membrane surfaces, which might have implications in the endocytosis processes [217], however the particular membrane recruitment mechanism is not known [218]. RNA stability and activity can be directly controlled by lipid membranes [5,6]; RNA degrades in the presence of lipid liquid membranes and the ribozymatic activity can be also controlled by various lipid species which might have implications *in vivo* (RNaseP, ribosomes, riboswitches stability and activity has vital role in cellular metabolism).

RNA–lipid interactions potentially play key roles in important biological processes such as viral replication and assembly. Membrane-enveloped RNA viruses – including SARS-CoV-2, HIV, and influenza – utilize host cell membranes as platforms for coordinated assembly [219,220]. For instance, Human Immunodeficiency Virus (HIV) assembly involves lipid binding and sorting steps and the formation of liquid-ordered membrane domains [175,176,221,222], which, in principle, could enhance the colocalization of HIV genomic RNA with sites of virus assembly and budding [12]. The precise cooperation among viral assembly components (lipids, proteins, and RNAs), including the establishment of specific interaction patterns (RNA-protein, protein-lipid, and possibly RNA–lipid interactions), is critical for viral infectivity. Although direct experimental evidence for RNA–lipid interactions during viral assembly is currently lacking, lipid-binding RNA motifs [215], including G-quadruplexes [5], are abundant in viral genomes [223], suggesting potential interactions. Furthermore, the influence of lipid membranes on naturally occurring ribozymes involved in viral genome replication, such as the Hepatitis Delta Virus (HDV) ribozyme [6], illustrates another mechanism by which lipid composition and membrane characteristics may influence virus fitness.

RNA–lipid interactions also hold promise for therapeutic approaches leveraging extracellular vesicles (EVs). These naturally derived lipid-enclosed carriers transport diverse biomolecules, including RNAs, while their intrinsic protein-lipid composition allows them to evade immune detection more efficiently than synthetic delivery systems. Upon uptake by recipient cells through endocytosis, EV-delivered molecules can modulate cellular metabolism and potentially facilitate horizontal gene transfer [224,225]. To fully exploit this therapeutic potential, a detailed understanding of EV cargo packaging specificity and membrane budding mechanisms is essential [226,227].

RNA–lipid interactions in modern biology are also an attractive subject due to development of mRNA vaccines. Recent events with the global pandemic of COVID-19 have proven that time plays a key role in vaccine development and the more this interaction is understood the quicker future mRNA–lipid nanoparticle-based vaccine development will be [228].

Most of the developed nucleic acid and lipid-based drugs and vaccines contain positively charged or ionizable lipid species (despite the possibility of RNA interaction with neutral lipids, such as DSPC, so far there was no success in the RNA cargo delivery in biological systems, thus the need for cationic lipid development emerged [229]). Such lipids directly interact with RNA and DNA molecules via electrostatic attraction, which makes them suitable systems for drug delivery. Usually the mix of cationic/ionizable lipids is used with neutral lipids, however single-component formulations were reported as well [230]. There is one limiting factor, which has slowed down development of such formulations: cationic lipids are toxic for cells and induce inflammatory response [231]. The presence of positive charge on the surfaces of RNA–lipid nanoparticles attract macrophages which quickly degrade the formulation. Attracted macrophages can also release cytokines which further increases the inflammatory response; alternatively, the presence of cationic formulations can also activate the complement system leading to inflammation [231–233]; cationic lipids can potentially interact with erythrocytes causing clotting and haemolysis [234]. Several approaches were taken in order to decrease toxicity of cationic lipids [235,236]. For example, in modern mRNA COVID vaccines, ionizable lipids become more positively charged only when uptake of the RNA–lipid nanoparticles by host cells takes place [237]. Ionizable lipids are cationic during formation allowing them to efficiently compartmentalize negatively charged mRNA. At neutral pH (around pH 7) lipids do not carry a positive charge, reducing nanoparticle toxicity. RNA–lipid nanoparticles then undergo endocytosis. The pH in the endosomes decreases and lipids become cationic which facilitates enhanced endosomal escape efficiency of mRNA [238–240]. Interestingly, the development of RNA–lipid formulations based on hydrogen bonding was reported as well [241]. It was proposed by several authors that, instead of using cationic lipids, it might be possible to use naturally existing non-toxic zwitterionic lipids [14,119,121]. Such a binding mechanism would, for example, rely on the presence of divalent ions, which might bridge phosphatidylcholine-RNA interactions. In modern mRNA vaccines, a cholesterol-DSPC mix is used as a lipid-based carrier together with cationic lipids; however, the presence of DSPC might be sufficient to carry nucleic acid cargo [123,128]. Further research on the topic might contribute to developing vaccines made of a simpler lipid mixfor example, based on zwitterionic gel membranes, which could increase vaccine shelf life and decrease potential handling costs.

## 9. Summary and future prospects

Despite growing knowledge about RNA–lipid interactions there are still a lot of details to reveal. To begin with, very little is known about the functional outcome of RNA–lipid binding. While some effects of lipids on RNA functionality and structure were reported, there is a large knowledge gap in this topic. It is not known how lipids can precisely influence RNA structure and how those changes might further bias RNA activity. Based on existing data, stabilization or destabilization of RNA-based structures is a double-edged sword and both effects might enhance, change, or inhibit RNA activity. Current results also show the effect of lipid membranes on RNA integrity which further extends the toolbox of RNA–lipid interactome. We propose that lipid species directly act on the RNA structure and depending on the lipid membrane composition and other features (for example, fluidity) different effects or RNA activity might be observed.

Interactions between lipids and RNA species are described as based on electrostatic and hydrophobic effects; however, the direct mechanism and structure of RNA–lipid complexes is still not well known. Current developments in molecular dynamic simulations confirm current findings about the importance of RNA sequence, structure, and lipid membrane fluidity for RNA–lipid interaction; however, it is still a challenge to simulate complex systems based on the RNA, lipids, and divalent ions due to the limitations of the used force fields.

Another challenge of investigating RNA–lipid interactions to overcome will be to understand how an RNA–lipid interactome can control cellular metabolism *in vivo*. It is tempting to assume that Earth’s life started as a protein-free RNA–lipid world which further evolved into modern cells. In this scenario it is plausible that vestigial remnants of the RNA–lipid world still play an important role. The presence of ribozymes, riboswitches, and aptamers in the living cells confirms that even simple RNA-based systems support life. So far, no lipid-sensitive riboswitches have been discovered in cells [242], thus its first discovery would significantly advance our understanding of the cellular interactome. Moreover, it will be crucial in the future to understand which RNAs are interacting with lipid membranes since it has been shown that some particular RNA species can co-localize with lipid membranes. Further functional characterization of RNA–lipid binding *in vivo* (i.e. RNA purpose, structure, cellular role), as well as *in silico* (structure prediction, lipid and RNA remodelling, binding mechanisms) would add tremendously to the still relatively young RNA–lipid scene.

Lastly, establishing a toolbox based on RNA–lipid interactions would provide a powerful yet simple approaches for synthetic biology, enabling scientists to partially recreate conditions relevant to the emergence of life and assemble primitive protocells.
